# Clinical and molecular characterization of the R751L-CFTR mutation

**DOI:** 10.1152/ajplung.00137.2020

**Published:** 2020-12-09

**Authors:** Iram J. Haq, Mike Althaus, Aaron Ions Gardner, Hui Ying Yeoh, Urjita Joshi, Vinciane Saint-Criq, Bernard Verdon, Jennifer Townshend, Christopher O’Brien, Mahfud Ben-Hamida, Matthew Thomas, Stephen Bourke, Peter van der Sluijs, Ineke Braakman, Chris Ward, Michael A. Gray, Malcolm Brodlie

**Affiliations:** ^1^Translational and Clinical Research Institute, Faculty of Medical Sciences, Newcastle University, Newcastle upon Tyne, United Kingdom; ^2^Paediatric Respiratory Medicine, Great North Children’s Hospital, Newcastle upon Tyne Hospitals NHS Foundation Trust, Newcastle upon Tyne, United Kingdom; ^3^Institute for Functional Gene Analytics, Department of Natural Sciences, Bonn-Rhein-Sieg University of Applied Sciences, Rheinbach, Germany; ^4^Cellular Protein Chemistry, Science4Life, Faculty of Science, Utrecht University, Utrecht, The Netherlands; ^5^Biosciences Institute, Faculty of Medical Sciences, Newcastle University, Newcastle upon Tyne, United Kingdom; ^6^Department of Paediatrics, West Cumberland Hospital, Whitehaven, United Kingdom; ^7^Respiratory Medicine, Royal Victoria Infirmary, Newcastle upon Tyne, United Kingdom

**Keywords:** CFTR mutations, cystic fibrosis, primary airway epithelial cells, R751L

## Abstract

Cystic fibrosis (CF) arises from mutations in the CF transmembrane conductance regulator (*CFTR*) gene, resulting in progressive and life-limiting respiratory disease. R751L is a rare CFTR mutation that is poorly characterized. Our aims were to describe the clinical and molecular phenotypes associated with R751L. Relevant clinical data were collected from three heterozygote individuals harboring R751L (2 patients with G551D/R751L and 1 with F508del/R751L). Assessment of R751L-CFTR function was made in primary human bronchial epithelial cultures (HBEs) and *Xenopus* oocytes. Molecular properties of R751L-CFTR were investigated in the presence of known CFTR modulators. Although sweat chloride was elevated in all three patients, the clinical phenotype associated with R751L was mild. Chloride secretion in F508del/R751L HBEs was reduced compared with non-CF HBEs and associated with a reduction in sodium absorption by the epithelial sodium channel (ENaC). However, R751L-CFTR function in *Xenopus* oocytes, together with folding and cell surface transport of R751L-CFTR, was not different from wild-type CFTR. Overall, R751L-CFTR was associated with reduced sodium chloride absorption but had functional properties similar to wild-type CFTR. This is the first report of R751L-CFTR that combines clinical phenotype with characterization of functional and biological properties of the mutant channel. Our work will build upon existing knowledge of mutations within this region of CFTR and, importantly, inform approaches for clinical management. Elevated sweat chloride and reduced chloride secretion in HBEs may be due to alternative non-CFTR factors, which require further investigation.

## INTRODUCTION

Cystic fibrosis (CF) is a life-limiting autosomal recessive condition arising from mutations in the gene encoding the CF transmembrane conductance regulator (CFTR) protein (1). This gives rise to multisystem disease involving chronic respiratory infection and inflammation, pancreatic exocrine insufficiency, and complications of CF, including CF-related diabetes, liver disease, and male infertility (2). In the lungs, defective chloride and bicarbonate transport by CFTR leads to airway surface dehydration and acidification (3–5). This results in impaired mucociliary clearance, chronic endobronchial infection, neutrophilic inflammation, and eventual respiratory failure (2, 6).

CFTR is a member of the ATP-binding cassette (ABC) transporter family. It comprises two homologous units each containing a transmembrane domain (TMD1 and TMD2) that form the ion channel pore and a nucleotide-binding domain (NBD1 and NBD2) connected by a regulatory (R) domain (7). The combined actions of R domain phosphorylation, ATP binding to the NBDs, and subsequent TMD conformational change result in CFTR activation and chloride secretion (8).

More than 2000 *CFTR* mutations have been identified to date, of which around 400 currently fulfill functional and clinical criteria for CF disease. Information regarding these mutations can be accessed from two databases: http://genet.sickkids.on.ca and the CFTR2 database at https://cftr2.org. Mutations can be broadly divided into six classes according to the specific defect in CFTR protein biology (9, 10). Class I mutations arise from nonsense, frameshift, or splicing mutations that produce premature termination signals or no mRNA, leading to absent protein production. Class II mutations occur as a result of misfolded CFTR protein, endoplasmic reticulum (ER)-associated degradation, and reduced CFTR trafficking to the cell surface. Although CFTR is expressed at the cell surface in class III mutations (e.g., the G551D mutation), defective nucleotide binding results in impaired channel gating. Class IV mutations arise from abnormalities in the channel pore resulting in restricted ion transport and conductance defects. Low levels of CFTR arise from splicing defects in class V mutations. Instability of CFTR at the cell surface results in high turnover and reduced levels of CFTR apical expression in class VI mutations (10).

Classifying the basic defect associated with CFTR mutations has facilitated a new therapeutic era in CF with the production of targeted small-molecule modulator therapies (11, 12). However, not all mutations are confined to individual classes, which poses additional challenges. This is exemplified by F508del, the most common CFTR mutation, where phenylalanine deletion at position 508 results in NBD1 instability and abnormal protein folding (class II), defective channel gating of CFTR that escapes ER processes (class III), and high turnover at the cell surface (class VI) (10). Classification is further complicated by the potential impact of modifier genes together with environmental, microbiological, and other non-CFTR factors.

R751L is a missense mutation involving arginine to leucine substitution at codon 751 within the R domain of CFTR arising from a G > T change at cDNA nucleotide position c.2252. R751L is poorly characterized; clinical information is limited to one reported case of an infant with pancreatic insufficiency and a sweat chloride of 73 mmol/L (http://genet.sickkids.on.ca), and there is no information regarding the underlying molecular defect. Two additional variants at position R751, R751C and R751P, are associated with respiratory disease (http://genet.sickkids.on.ca), but again detailed information is lacking.

The specific location of R751L could give rise to a number of potential functional defects. First, proximity to the consensus phosphorylation site Serine 753 (S753) and other sites interacting with the NBDs and COOH terminus may reduce CFTR function (13–15). Second, mutation of the methylation site Arg^751^ could impact upon posttranslational modification of CFTR, as proposed for R751P and R751C (16). Third, ATP binding and channel gating may be affected by interactions of residues 748 to 778 with NBD1 (13). Finally, loss of positive charge arising from arginine to leucine substitution may impact upon CFTR instability.

In the current era of CFTR modulator therapy, there is an essential need to understand the molecular and functional implications of rare mutations to correctly inform the most appropriate therapies. Here, we have identified a family of three individuals carrying two CFTR mutations, of which one is R751L, and describe their clinical phenotype. We provide a molecular characterization of R751L-CFTR and the resultant impact on CFTR function using an in vitro expression system and an ex vivo cell culture model derived from a heterozygote patient with R751L and the severe disease-causing F508del mutation.

## MATERIALS AND METHODS

### Study Subjects and Ethical Approvals

This study involved retrospective data collection from patient medical records with relevant written permissions. Primary human bronchial epithelial (HBE) cells were collected from children with and without CF undergoing a clinically indicated bronchoscopy with written informed consent and relevant ethical approval (UK National Research Ethics Committee 15/NE/0215).

### Primary Cell Culture

HBEs were collected by bronchoscopic brushing of the mucosal surface of a second- or third-generation bronchus. First-passage HBEs were seeded on collagen-coated Transwell inserts (Corning, Sigma-Aldrich, Dorset, UK) and maintained in an air-liquid interface (ALI) culture for 3 to 4 wk. Fully differentiated HBEs were characterized through demonstration of mucus production, cilia formation, and transepithelial resistance (R_t_) values >300 Ω.cm^2^ (STX2 electrodes, EVOM2TM Epithelial Voltohmmeter; World Precision Instruments, Hertfordshire, UK).

### Short-Circuit Current Assessment

To assess CFTR function, differentiated first-passage HBE ALI cultures were mounted in Ussing chambers (Physiologic Instruments, San Diego, CA) to measure short-circuit currents (*I_sc_*). HBEs were equilibrated in symmetrical apical and basolateral Krebs buffer solution (containing in mM: 115 NaCl, 25 NaHCO_3_, 5 KCl, 1 CaCl_2_, and 1 MgCl_2_, pH 7.4, 37°C) and gassed with 5% CO_2_-95% O_2_. The following reagents (all from Tocris Bioscience, Abingdon, UK) were sequentially added to the apical compartment and corresponding short-circuit current (*I_sc_*) changes recorded: amiloride (100 µM) to inhibit the epithelial sodium channel (ENaC), forskolin (10 µM) to stimulate CFTR, and CFTR_inh_-172 (20 µM) to inhibit CFTR. Data were recorded and digitized using Acquire and Analysis software (version 2.3; Physiologic Instruments, San Diego, CA).

### Heterologous Expression of cRNA in Xenopus Oocytes and Two-Electrode Voltage Clamp Recordings

#### Synthesis of cRNA and heterologous expression in Xenopus oocytes.

The R751L-CFTR mutation was generated by site-directed mutagenesis using the QuikChangeLightning Kit (Agilent Technologies, Cheshire, UK) according to the manufacturer’s instructions and using the forward (5′-ctgatcacgctgatgagaggcagtatcgcct-3′) and reverse primer (5′-aggcgatactgcctctcatcagcgtgatcag-3′) sequences. Successful mutation generation was verified by DNA sequencing and cRNA synthesized as previously described (17). Plasmid DNA containing human CFTR was kindly provided by Professor Blanche Schwappach (University of Goettingen).

Procedures using *Xenopus* tissues were approved by the Newcastle University Animal Welfare and Ethical Review Body (project ID 630). *Xenopus laevis* ovaries (European *Xenopus* Resource Center, Portsmouth, UK) were dissected and incubated for 90 min in 2.6 mg/mL collagenase A (Roche, West Sussex, UK) at room temperature in ORII solution (containing in mM: 82.5 NaCl, 2 KCl, 1 MgCl_2_ × 6H_2_O, and 10 HEPES, pH 7.5). The cell suspension was washed with equal volumes of ORII and Modified Barth’s solution (MBS in mM: 88 NaCl, 1 KCl, 2.4 NaHCO_3_, 0.82 MgSO_4_ × 7H_2_O, 0.33 Ca(NO_3_)_2_ × 4H_2_O, 0.41 CaCl_2_ × 2H_2_O, 10 HEPES, 20 µg/mL gentamycin, pH 7.5). Stages V and VI oocytes were stored at 16°C in MBS. Fifty-one nanoliters of cRNA was injected into oocytes with a Nanolitre-Injector (Drummond Scientific, Broomall, PA), yielding final concentrations of 12.5 ng RNA/oocyte. Injected oocytes were cultured for 3 days at 16°C in MBS.

Oocytes were placed in a Lucite chamber and continuously perfused with oocyte Ringer’s solution (in mM: 90 NaCl, 1 KCl, 2 CaCl_2_, and 5 HEPES, pH 7.4). Microelectrode recordings were performed as previously described (17). Transmembrane currents (*I_M_*) were recorded at a holding potential of −60 mV, low-pass filtered at 1 kHz (LPF-202; Warner Instruments, Hamden, CT), and recorded by a strip chart recorder (Kipp & Zonen, Delft, The Netherlands). Experiments were designed to investigate maximal differences in CFTR activation, where *I_M_* responses to forskolin (5 µM), a CFTR potentiator, genistein (40 µM), and CFTR_inh_-172 (10 µM; Tocris Bioscience, Abingdon, UK) were recorded and digitized using Inkscape (version 0.91). All recordings were performed at room temperature.

### Pulse Chase Experiments and Transport Assays in Human Embryonic Kidney 293 Cell Transfectants

Human embryonic kidney 293 (HEK-293) cells were transfected with pBi.CMV2-CFTR constructs harboring the R751L-CFTR mutant, as described below. Cells were used after 24 h for radioactive pulse chase-limited proteolysis experiments or cell surface biotinylation assays in the presence of known CFTR modulators as described previously (18, 19) and below.

#### HEK-293 cell culture and transient expression.

HEK-293 cells were maintained in DMEM (Thermo Fisher Scientific, Paisley, UK) supplemented with 10% FCS (qualified, E.U. approved, South America origin) and 2 mM Glutamax (Thermo Fisher Scientific, Paisley, UK) at 37°C and 5% CO_2_. Cells were grown in 6 cm^2^ dishes to ∼70% confluency and transfected using Lipofectamine 3000 (Thermo Fisher Scientific, Paisley, UK) according to the manufacturer’s instructions or PEI MAX40 (Polysciences, Hirschberg an der Bergstrasse, Germany) as previously described (19). The R751L-CFTR mutant was made by Gibson assembly (New England Biolabs, Leiden, The Netherlands) using the template pBi.CMV2-CFTR (19) and the following primers: 5′-gaggcgatactgcctctcatcagcgtgatcagc-3′, 5′-tgctgatcacgctgatgagaggcagtatcgcctc-3′, 5′-taaccctgataaatgcttcaataatattgaaaaaggaagagt-3′, and 5′-agcatttatcagggttattgtctcatgagc-3′.

#### Pulse-chase analysis and limited proteolysis.

HEK-293 cells were used 24 h posttransfection for pulse-chase experiments as described (18, 19). Briefly, cells were labeled for 15 min with 143 µCi/6 cm^2^ dish of EasyTag Express ^35^S-protein-labeling mix (Perkin Elmer, Groningen, The Netherlands). Labeling was stopped by adding excess unlabeled cysteine and methionine, and the labeled protein was chased for different periods of time. Where indicated, CFTR modulators VX-770 or VX-809 (Selleckchem, Houston, TX) were added to 3 µM (final concentration) in media during starvation, pulse, and chase. Dishes were transferred to ice and washed twice with ice-cold HBSS (Life Technologies, Bleiswijk, The Netherlands). Cells were lysed using 1% Triton X-100 in a mixed buffer containing (in mM) 20 MES, 100 NaCl, and 30 Tris·HCl, pH 7.5 (MNT) (all from Sigma-Aldrich, Zwijndrecht, The Netherlands). Lysates were cleared by centrifugation for 10 min at 16,000 *g* and 4°C. The supernatant was used for limited proteolysis (see below) or direct immunoprecipitation and analysis using 7.5% or 12% SDS-PAGE (19, 20). In brief, detergent lysates were incubated with 25 µg/mL proteinase K from tritirachium album (Sigma-Aldrich, Zwijindrecht, The Netherlands) for 15 min on ice. Digestions were stopped by adding an equal volume of MNT, 1% Triton X-100, 2 mM PMSF, and 2 µg/mL of chymostatin, leupeptin, antipain, and pepstatin A (all from Sigma-Aldrich, Zwijndrecht, The Netherlands), and fragments were analyzed using domain-specific antibodies.

#### Immunoprecipitation.

Full-length CFTR in nontreated lysates and limited-proteolysis samples were transferred to 50 µL of protein-A beads (GE Healthcare Life Sciences, Eindhoven, The Netherlands) with pre-adsorbed antibody and immunoprecipitates as described (19). Rabbit antibodies Mr. Pink (against NBD1), transmembrane domain (TMD) 1C (against TMD1), TMD2C (against TMD2), and mouse monoclonal 596 (against NBD2) were previously described (19); 596 was kindly provided by Dr. John Riordan (University of North Carolina School of Medicine) (21).

All immunoprecipitates were washed twice for 10 min at room temperature. Proteolytic fragments originating from TMD1 were immunoprecipitated for 3 h at 4°C with TMD1-C antibody and washed with 10 mM Tris·HCl, pH 8.6, 300 mM NaCl, 0.05% SDS, and 0.05% Triton X-100. NBD1 fragments were immunoprecipitated overnight at 4°C with Mr. Pink antibody and washed with 10 mM Tris·HCl, pH 8.6, 300 mM NaCl, 0.1% SDS, and 0.05% Triton X-100. Fragments derived from TMD2 were immunoprecipitated for 3 h at 4°C with TMD2-C antibody and washed with 50 mM Tris·HCl, pH 8.0, 150 mM NaCl, and 1 mM EDTA. NBD2-derived fragments were immunoprecipitated for 3 h at 4°C with 596 antibody and washed using 30 mM Tris·HCl, pH 7.5, 20 mM MES, 100 mM NaCl, and 0.5% Triton X-100. Washed immunoprecipitates were resuspended in 10 µL of 10 mM Tris·HCl, pH 6.8, plus 10 µL of 2× reducing Laemmli sample buffer and denatured for 5 min at 55°C before analysis by 12% SDS-PAGE. Gels were dried and exposed to X-ray film for display.

#### Cell-surface biotinylation and Western blot.

The pool of plasma membrane CFTR was assayed by cell-surface biotinylation, followed by neutravidin pulldown and Western blot, as previously described (19). In brief, cells received fresh medium with VX-770 or VX-809 (3 µM) 4 h after transfection and were maintained for 16 h. Dishes were transferred on ice and washed twice with PBS^++^. Next, dishes received 500 µL of PBS^++^ containing 0.5 mg/mL sulpho-NHS-SS-Biotin (Thermo Fisher Scientific, Bleiswijk, The Netherlands) and were incubated for 30 min on ice. Nonreacted sulpho-NHS-SS-Biotin was quenched, and cells were lysed in 300 µL of MNT, 1% Triton X-100, 1 µg/mL of chymostatin, leupeptin, antipain, and pepstatin A, and 1 mM PMSF (all from Sigma-Aldrich, Zwijndrecht, The Netherlands). Lysates were cleared by centrifugation for 10 min at 16,000 *g* and 4°C. An aliquot of 10 µL of lysate was transferred to an equal volume of 2× reducing Laemmli sample buffer and saved as input sample. The remaining lysate was incubated with 25 µL of Neutravidin beads (Thermo Fisher Scientific, Paisley, UK) for 1 h at 4°C and washed twice with 10 mM Tris·HCl, pH 8.6, 300 mM NaCl, 0.1% SDS, and 0.05% Triton X-100 (all from Sigma-Aldrich, Zwijndrecht, The Netherlands). Beads were resuspended in 15 µL of 10 mM Tris·HCl, pH 6.8, and 15 µL of 2 × reducing Laemmli sample buffer, and bound proteins were eluted and incubated for 5 min at 55°C. Samples were resolved on 7.5% SDS-PAA gels and analyzed by Western blot using monoclonal antibody 596 (1:5,000), rabbit anti-actin (1:5,000; Sigma-Aldrich, Paisley, UK), and goat-anti-mouse Alexa-800 (1:10,000; LI-COR, Hamburg, Germany) and donkey-anti-rabbit Alexa-680 (1:10,000; LI-COR) secondary antibodies. Detection was performed using infrared imaging (Odyssey CLx; LI-COR, Hamburg, Germany).

### Statistical Analysis

Results are presented as individual data points with the mean (SD) for HBE culture inserts or *Xenopus* oocytes. Normality of data was assessed with Shapiro-Wilk testing. Statistical analysis was performed using an unpaired *t* test (unless specified otherwise) in GraphPad Prism software (version 8.2; GraphPad, San Diego, CA). *P* values of <0.05 were considered statistically significant.

## RESULTS

### Individuals with R751L-CFTR Display a Mild Disease Phenotype

We identified three individuals with one R751L allele within the same family. None of the patients were on CFTR modulator therapies, and R751L was not linked to another CFTR variant in *cis*. Here, we present a clinical description for each individual (summarized in Table 1).

**Table 1.** Clinical summary of study individuals and F508del/F508del participants

Parameter	*Patient 1*	*Patient 2*	*Patient 3*	F508del/F508del CF Patients (*n *=* *3)
Age at assessment, yr	8	26	35	Mean 7.7 (range 5.7–11.4)
Sex (M/F)	M	F	M	M: 1F: 2
CFTR genotype	F508del/R751L	G551D/R751L	G551D/R751L	F508del/F508del
Sweat chloride, mmol/L	71–88	73–100	80–100	107 ± 6.3 (mean ± SD)
Pancreatic status	Sufficient	Sufficient	Sufficient	Insufficient
Nutritional status	Height and weight: 91^st^ centiles	BMI (kg/m^2^): 39	BMI (kg/m^2^): 23	
Radiological findings	CXR - normal	CXR - normal	CXR - normal	CXR − bronchial wall thickening (2/3)CT − bronchiectasis (1/3)
Recent FEV_1_, %predicted	116	106	92	94.6 (range 86 to 100)
Respiratory microbiology	*B. cepacia* *P. aeruginosa*	*P. aeruginosa*	*H. influenzae*	*H. influenzae * *S. pneumoniae * *A. fumigatus * *P. aeruginosa * *Parainfluenza virus*

BMI, body mass index; CT, computerized tomography; F, female; FEV_1_, forced expiratory volume in 1 s; M, male.

#### Patient 1 (F508del/R751L).

*Patient 1* was a Caucasian male born in 2011. Immunoreactive trypsin (IRT) was elevated on newborn screening with identification of F508del on subsequent DNA analysis. Repeat IRT at 1 mo remained elevated. In 2016, R751L was identified as the second allele following an extended screen of 50 CFTR mutations.

As a young infant, initial sweat chloride was elevated (74 mmol/L), with evidence of mild pancreatic insufficiency (fecal elastase 110 µg/g). A repeat investigation at 1 yr showed an elevated sweat chloride (88 mmol/L) and improved exocrine pancreatic function (487 µg/g) and pancreatic sufficiency. Growth was well maintained with height and weight parameters following the 91st centiles. *Burkholderia cepacia* was isolated on a respiratory culture in 2016 following persistent respiratory symptoms despite standard antimicrobial therapy. The patient remained clinically well thereafter with regular mucolytic administration and physiotherapy for low-grade sputum production. *Pseudomonas aeruginosa* was isolated on a routine respiratory culture in 2019 and treated with targeted antimicrobial therapy. Annual chest X-ray (CXR) surveillance did not show features of respiratory disease. and recent lung function assessment with forced expiratory volume in 1 s (FEV_1_) was 116% predicted.

#### Patient 2 (G551D/R751L).

*Patient 2* (aunt to *patient 1*) was a Caucasian female diagnosed with CF in 1993 at 6 mo of age following elevated sweat chlorides (73–100 mmol/L) and persistent respiratory symptoms. Subsequent DNA analysis revealed the G551D CFTR mutation. The second mutation was identified as R751L with extended mutation analysis in 2016. IRT was negative, and the patient remained pancreatic sufficient with normal fecal elastase. *P. aeruginosa* was isolated in 2003 but eradicated with antimicrobial treatment. *Patient 2* has remained well without treatment with recent predicted FEV_1_ of 106% and normal CXR findings.

#### Patient 3 (G551D/R751L).

*Patient 3* (uncle to *patient 1*, brother to *patient 2*) was a Caucasian male born in 1984 and was diagnosed with CF following the diagnosis of *patient 2* in 1993. Initial DNA analysis revealed G551D as one of the CFTR mutations, and R751L was identified on extended mutation analysis in 2016. Sweat chloride values were elevated (80–100 mmol/L), and respiratory microbiology included one *Haemophilus influenzae* isolate that was successfully treated. Semen analysis showed normal sperm function, and additional medical history included recurrent episodic pancreatitis. The patient remained clinically well without treatment with normal CXR findings and recent predicted FEV_1_ of 92%.

### F508del/R751L HBEs Have Reduced but Functional CFTR Activity

**Figure 1.**
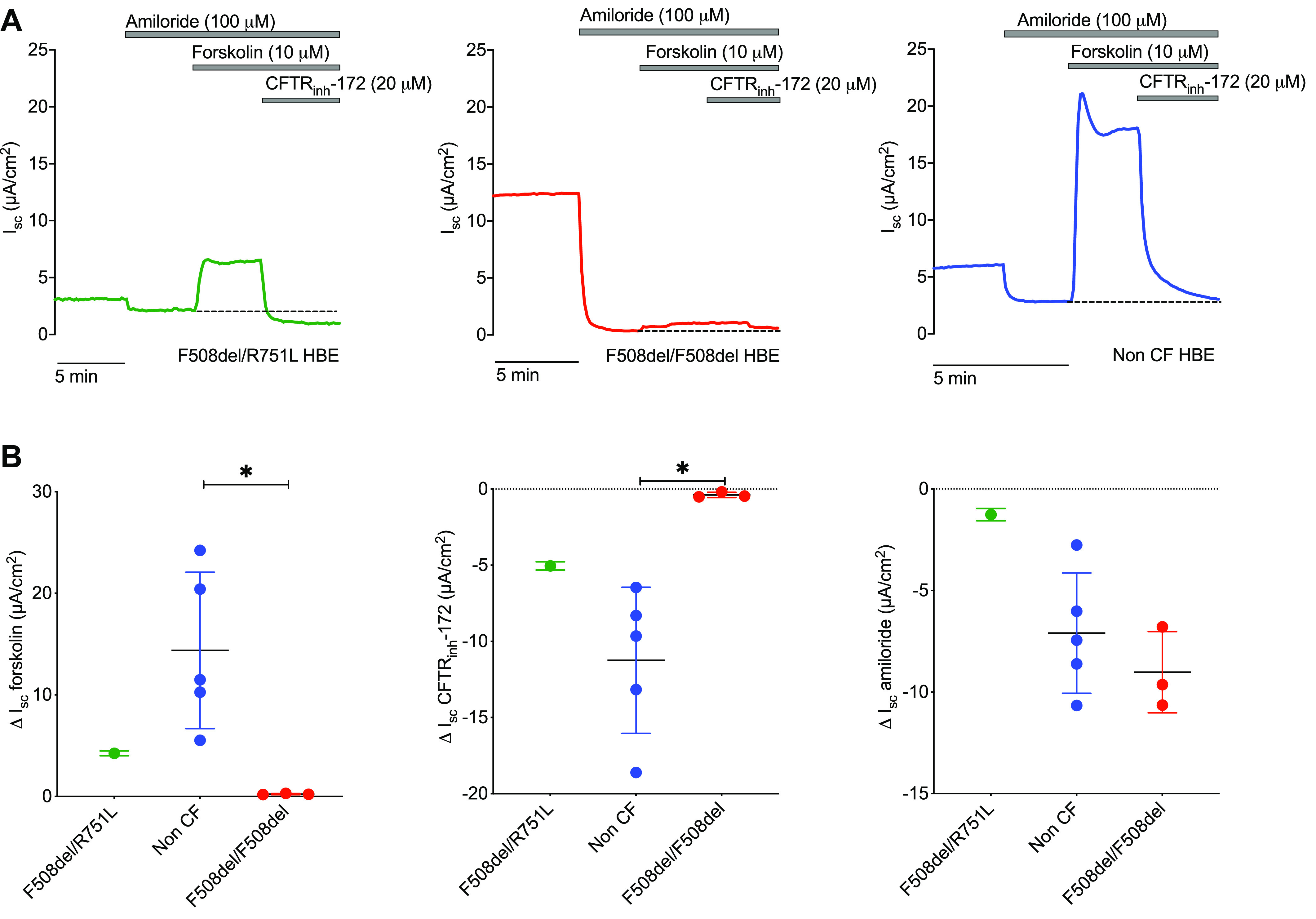
Short-circuit current responses in F508del/R751L, non-cystic fibrosis (CF), and F508del/F508del human bronchial epithelial (HBE) cultures. *A*: short-circuit current (*I_sc_*) responses to amiloride (100 µM), forskolin (10 µM), and CF transmembrane conductance regulator (CFTR)_inh_-172 (20 µM) were measured using Ussing chamber experiments in F508del/R751L, non-CF, and F508del/F508del HBEs. Representative traces shown from *n* = 1 donor in each graph. Lines indicate reagent addition to the apical Ussing chamber, which remained present in solution throughout the recordings. Functional CFTR is represented by a positive *I_sc_* deflection arising from net anion basolateral-apical secretion following forskolin addition. Dashed line indicates the baseline *I_sc_* prior to forskolin addition. CFTR is inhibited by the addition of CFTR_inh_-172. Amiloride addition inhibits epithelial sodium channel (ENaC)-mediated sodium absorption, resulting in a downward *I_sc_* deflection. *B*: comparative assessment of *I_sc_* showed greater responses to forskolin and CFTR_inh_-172 in non-CF compared with F508del/R751L HBEs. F508del/R751L HBEs demonstrated the smallest response to amiloride. Data are presented as mean (SD) responses for each donor and analyzed using unpaired *t* test. F508del/R751L HBEs were not included in statistical comparisons; *n* = 1 F508del/R751L donor, *n* = 5 non-CF donors, and *n* = 3 F508del/F508del donors for each graph. Minimum 3 culture inserts used from each donor. *P < 0.05.

To investigate CFTR function associated with F508del/R751L, we measured *I_sc_* responses in differentiated primary HBEs derived from *patient 1*. In this system, functional CFTR is represented by a positive *I_sc_* deflection arising from net anion basolateral-apical secretion. After ENaC inhibition with amiloride, forskolin-mediated cAMP elevation resulted in CFTR activation (Fig. 1*A*). This response was inhibited by CFTR_inh_-172, confirming the presence of functional CFTR. The mean CFTR_inh_-172-sensitive *I_sc_* was 15% greater than the forskolin response, suggesting basal CFTR activity in the absence of forskolin.

Responses were compared with HBEs derived from children without CF and three F508del homozygous children (clinical profiles summarised in Table 1), the latter as a comparator of a CFTR mutation associated with developing severe disease. Representative *I_sc_* responses from non-CF and F508del/F508del HBEs are shown in Fig. 1*A*.

The forskolin-induced *I_sc_* was greater in F508del/R751L HBEs compared with F508del/F508del HBEs but 71% smaller than the mean forskolin-induced *I_sc_* derived from non-CF HBEs (Fig. 1*B*). These differences were paralleled in responses to CFTR_inh_-172, where mean F508del/R751L HBE responses were 55% smaller than those in non-CF HBEs and greater than those in F508del/F508del HBEs. The amiloride-sensitive *I_sc_* was lowest in F508del/R751L HBEs compared with both non-CF and F508del/F508del HBEs. As expected, F508del/F508del HBE responses to forskolin and CFTR_inh_-172 were significantly lower than those seen with non-CF HBEs due to the lack of functional CFTR (*P* < 0.05).

These findings demonstrate residual CFTR function in F508del/R751L HBEs, which was smaller in magnitude than in non-CF HBEs. ENaC current was reduced in F508del/R751L HBEs compared with non-CF and F508del/F508del HBEs.

### R751L CFTR-Expressing Xenopus Oocytes Demonstrate CFTR Activity

**Figure 2.**
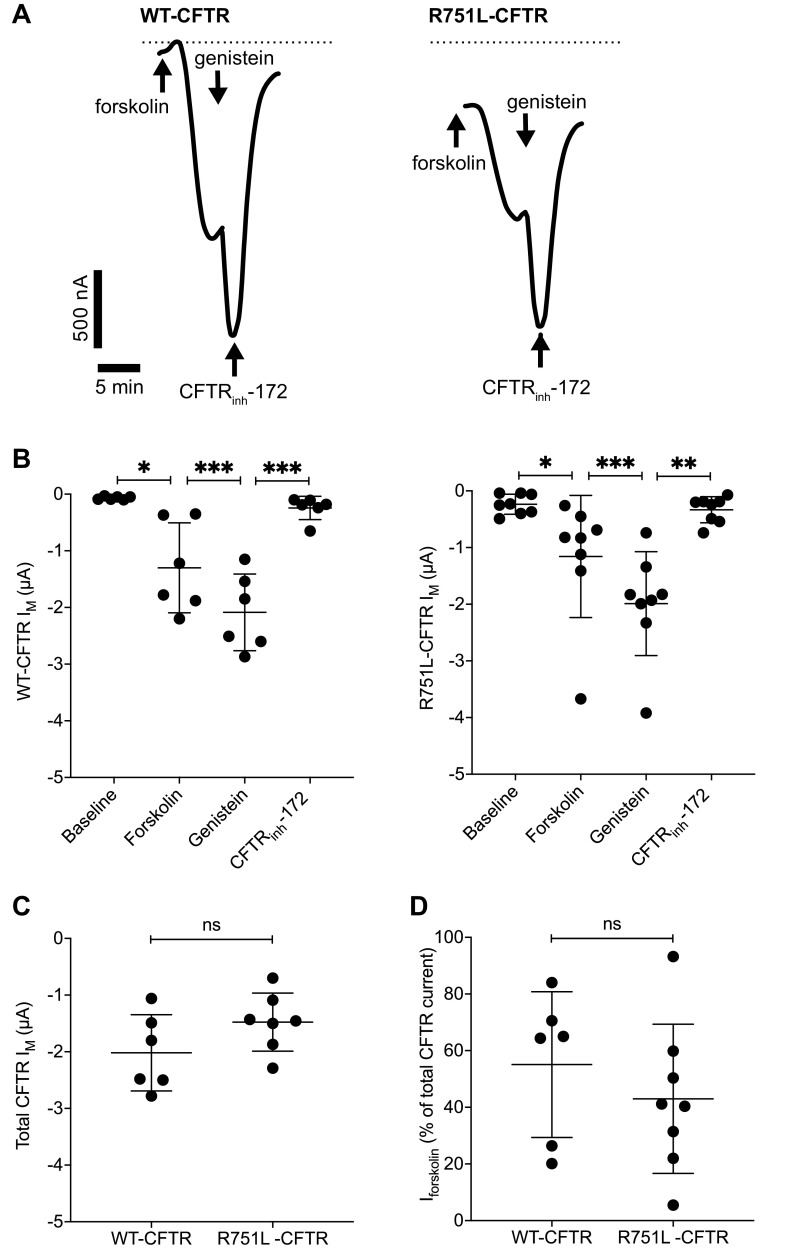
Transmembrane currents in wild-type (WT) and R751L-CFTR-expressing *Xenopus* oocytes. *A*: transmembrane current (*I_M_*) responses to forskolin (5 µM), genistein (40 µM), and cystic fibrosis transmembrane conductance regulator (CFTR)_inh_-172 (10 µM) were measured in *Xenopus* oocytes expressing WT and R751L-CFTR, with representative traces shown from *n* = 1 oocyte. Experiments were performed at room temperature. Arrows indicate reagent addition to the bathing solutions, with evidence of a time lag between reagent addition and response. Downward deflections in *I_M_* are indicative of anion efflux. Dashed lines indicate zero current for representative recordings. *B*: *I_M_* measurement in oocytes expressing WT and R751L-CFTR demonstrated forskolin and genistein-induced *I_M_*, which were inhibited by CFTR_inh_-172. Peak *I_M_* values depicted after drug application from individual experiments with corresponding mean (SD). Data were analyzed using paired *t* test to demonstrate that added reagents affected the resultant *I_M_*. *C*: comparative assessment of total CFTR *I_M_* (the sum of forskolin and genistein-induced *I_M_*) was the same in WT and R751L-CFTR-expressing oocytes. Peak *I_M_* values depicted after drug application from individual experiments with corresponding mean (SD). Data analyzed using unpaired *t* test. *D*: the forskolin fraction of the CFTR current (%total CFTR current) was the same in WT and R751L-CFTR-expressing oocytes. Data were analyzed using unpaired *t* test; *n* = 6 WT-CFTR and *n* = 8 R751L-CFTR-expressing oocytes. *P < 0.05; **P < 0.01; ***P < 0.001. ns, not significant.

To further investigate CFTR activity associated with R751L in isolation, *I_M_* responses to forskolin and genistein were measured in R751L-CFTR-expressing *Xenopus* oocytes, with wild-type (WT)-CFTR-expressing oocytes as a positive control. Representative *I_M_* traces for WT-CFTR and R751L-CFTR are shown in Fig. 2*A*. In both WT and R751L-CFTR-expressing oocytes, forskolin induced an *I_M_* increase, as indicated by the downward deflection. This was further augmented with genistein and inhibited with CFTR_inh_-172 (Fig. 2*B*).

Comparative assessment of the total CFTR current (*I_M_* induced by forskolin and genistein) did not reveal significant differences between WT and R751L-CFTR (Fig. 2*C*). Furthermore, there was no difference between WT and R751L-CFTR in relative stimulation of channel activity by forskolin with respect to total currents (Fig. 2*D*).

These findings confirm functional CFTR activity associated with R751L, with a magnitude analogous to WT-CFTR in a heterologous expression system.

### R751L Is Transported to the Golgi Complex like WT-CFTR

**Figure 3.**
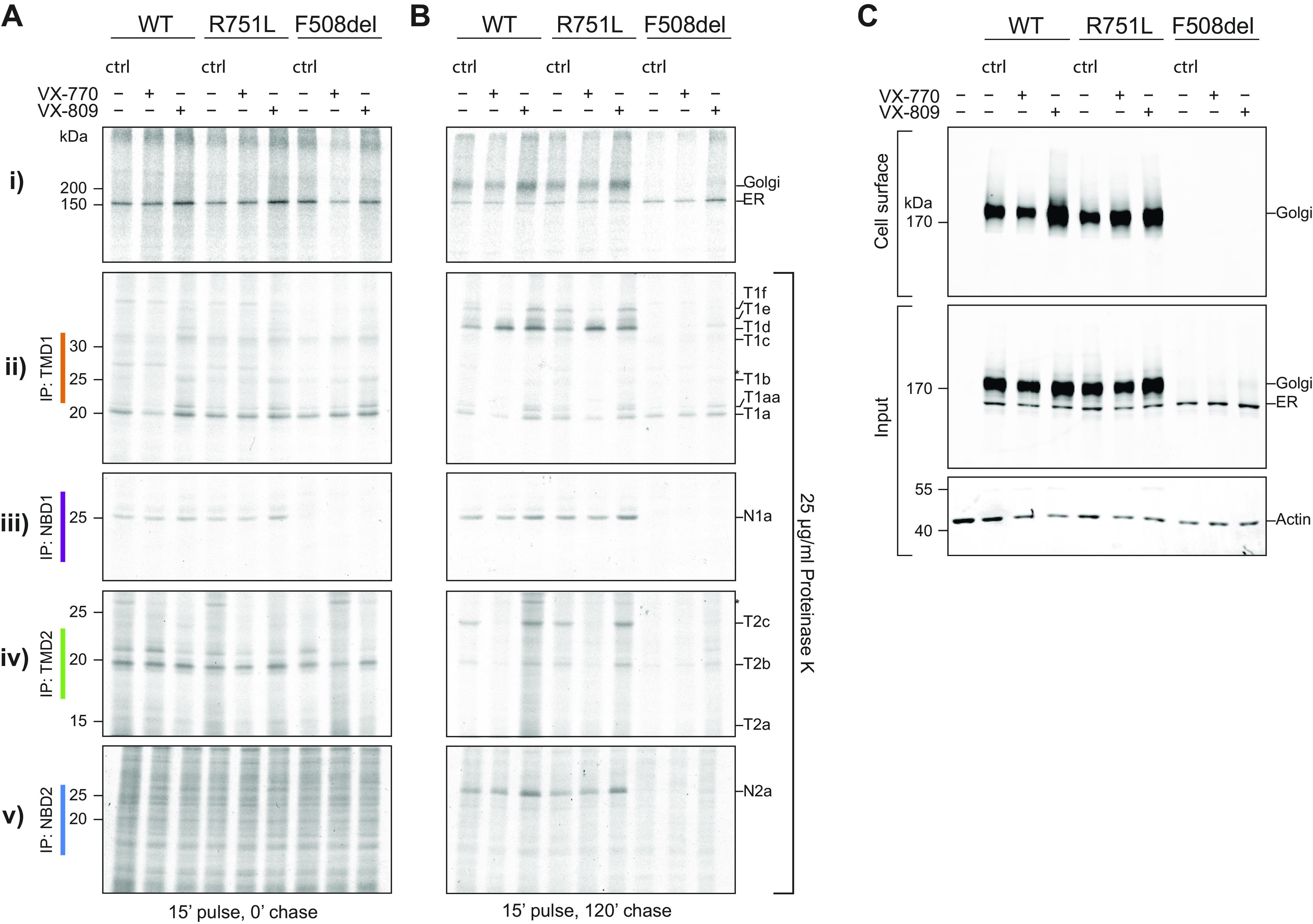
Impact of R751L on cystic fibrosis transmembrane conductance regulator (CFTR) protein folding and transport and cell surface expression. *A* and *B*: *i*) human embryonic kidney 293 (HEK-293) cells expressing wild-type (WT), R751L, and F508del CFTR constructs were labeled with ^35^S-methionine/cysteine for 15 min and chased for 0 (0′; *A*) and 2 h (120′; *B*) in the presence of VX-770 (3 µM), VX-809 (3 µM), or DMSO control (ctrl). Cells were lysed in 1% Triton X-100, and lysates were treated or not with proteinase K (25 µg/mL) for 15 min. CFTR and fragments were immunoprecipitated using TMD1C (TMD1; *ii*), Mr. Pink (NBD1 and full-length CFTR; *iii*), TMD2C (TMD2; *iv*), or 596 (NBD2) antibodies (*v*). *Nonspecific bands. N1a, protease-resistant NBD1-specific fragment; N2a, protease-resistant NBD2-specific fragment; T1d-f, protease-resistant TMD1-specific fragments; T2c, protease-resistant TMD2-specific fragment. T1, T2, and N2 fragments represent domain assembly, which is a late-folding stage of CFTR. *C*: cell surface biotinylation was performed in HEK-293 cells expressing CFTR constructs in the presence of VX-809, VX-770, or DMSO ctrl pretreatment. Cells were lysed in 1% Triton X-100, and lysates were used for pulldown of biotinylated proteins with Neutravidin beads. Proteins were analyzed on 7.5% SDS-PAA gels and transferred to PVDF membrane and blotted for CFTR (596) or actin. R751L was similar to WT-CFTR in transport to the Golgi (120′ chase), protein folding (protease resistance) of all 4 domains at both time points, and presence at the cell surface. Quantification for this data from 4 independent experiments is shown in Fig. 4.

In light of these findings, we next assessed the transport of R751L in the secretory pathway. HEK-293 cells expressing R751L-CFTR, WT-CFTR (positive control), and F508del-CFTR (negative control) were labelled with ^35^S-methionine/cysteine during a 15-min pulse (Fig. 3*A*), and the radiolabelled protein was chased in the presence of excess unlabeled amino acids (Fig. 3*B*). Detergent cell lysates were subjected in parallel with immunoprecipitation of CFTR (Fig. 3, *A* and *B*, *panel i*) and limited proteolysis (Fig. 3, *A* and 3*B* *panels ii–iv*), as previously detailed (19, 20, 22).

In the 15-min pulse time, both mutants were synthesized to similar levels as WT-CFTR (Fig. 3*A*, *panel i*, control lane). During the chase, both WT and R751L-CFTR completed folding and traveled to the Golgi complex, where the ER forms changed into the Golgi forms due to glycan modifications (Fig. 3*B*, *panel i*, control lane). The F508del mutation instead caused CFTR misfolding and degradation, leading to absence of the Golgi form and a decrease in ER form to 68 ± 13% (*n* = 3).

These findings demonstrated that the R751L-CFTR mutant reached the Golgi as efficiently as WT-CFTR.

### Folding of CFTR Is Not Affected by R751L

To assess folding of R751L-CFTR, detergent lysates were subjected to limited proteinase K digestion (25 µg/mL for 15 min on ice), followed by immunoprecipitation with CFTR-domain-specific antibodies. Using this technique, misfolded proteins demonstrate increased protease susceptibility, which can be detected by SDS-PAGE (19, 20, 22).

Immediately after synthesis, the major fragments of the first transmembrane domain, TMD 1 (T1a-c), and the second TMD, TMD2, (T2b), were the same for R751L-CFTR, WT-CFTR, and F508del-CFTR, suggesting similar folding of both TMDs in all three proteins (Fig. 3*A*, *panels ii* and *iv*, control lanes). Whereas R751L NBD1 folding was similar to WT NBD1 (fragment N1a), F508del NBD1 was completely digested (Fig. 3*A*, *panel iii*, control lanes). NBD2 was not resistant to digestion immediately after synthesis, and because of epitope absence, the antibody demonstrated increased immunoprecipitated background for all three constructs (Fig. 3*A*, *panel v*, control lanes).

At 2 h (120′ chase), R751L was still indistinguishable from WT-CFTR; TMD1, TMD2, and NBD2 became more protease resistant (Fig. 3*B*, *panels i*, *ii*, *iv*, and *v*, control lanes), yielding larger fragments, whereas NBD1 remained folded (Fig. 3*B*, *panel iii*, control lanes).

The sensitivity of each construct to VX-770 or VX-809 was examined by modulator addition from 15 min before pulse labeling to the end of the chase. VX-809 rescued some F508del CFTR from the ER, since after 2 h of chase a Golgi band was visible (Fig. 3*B*, *panel i*). NBD1 was not rescued, but the other domains showed a small increase in protease resistance (Fig. 3*B*, *panels ii*–*iv*) (19). Both R751L and WT-CFTR showed a small increase in expression upon VX-809 treatment, also yielding an increase in the Golgi form (Fig. 3*B*, *panel i*). The effects of VX-770 on WT and R751L-CFTR were also the same (Fig. 3*B*, *panel i*), demonstrating a small, destabilizing effect on TMD1 (decrease of T1e and T1f) and TMD2 (decrease of T2b and T2c) (Fig. 3*B*, *panels ii*–*v*) (19).

### R751L and WT-CFTR Show Similar Cell Surface Localization

To establish whether the Golgi form of R751L-CFTR reached the cell surface, we compared this mutant with WT and F508del-CFTR (Fig. 3*C*). Cell-surface biotinylation showed that only the Golgi form of CFTR was detectable on the cell surface, with no difference between WT and R751L-CFTR (Fig. 3*C*, control lanes). F508del-CFTR did not reach the cell surface detectably, either with or without VX-809.

**Figure 4.**
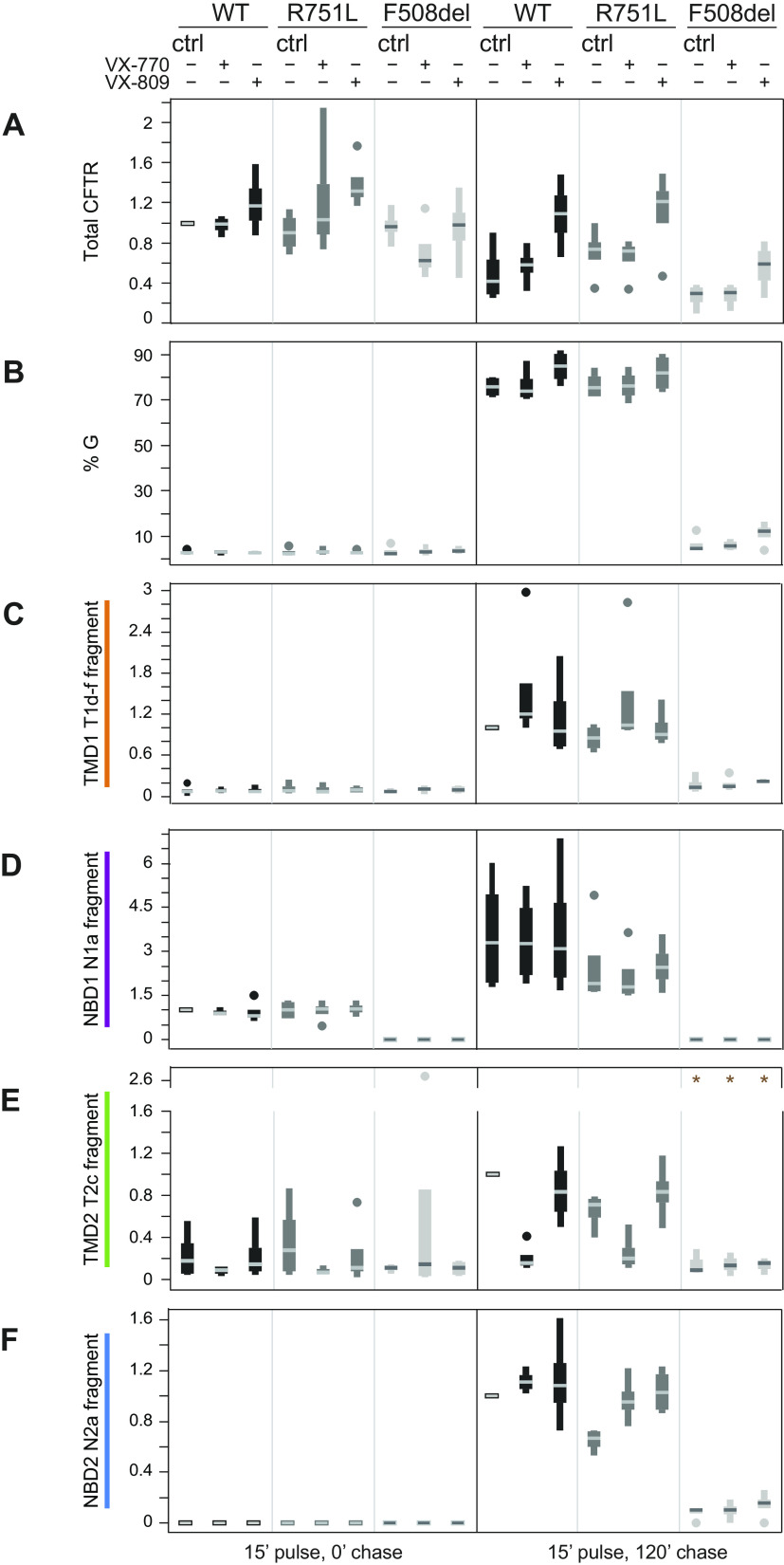
Quantification of total cystic fibrosis transmembrane conductance regulator (CFTR), %Golgi-modified form, and CFTR domain-specific fragments as shown in Fig. 3. *A*–*F*: quantification data for 4 independent experiments together with mean and SD values for the following. *A*: fold increase in the amount of total CFTR [endoplasmic reticulum form of CFTR (ER) + complex-glycosylated Golgi form of CFTR which has left the ER and resides in or beyond the Golgi complex including the plasma membrane (G)] relative to wild-type (WT)-CFTR. *B*: %Golgi-modified form [G/(ER + G). *C*–*F* shows the fold increase in the amount of folded CFTR domain-specific fragments relative to WT-CFTR in DMSO as follows: TMD1 (T1d-f/(ER + G) (*C*), NBD1 (N1a/(ER + G) (*D*), TMD2 (T2c/(ER + G) (*E*), and NBD2 (N2a/(ER + G) (*F*). Data in *A* and *D* have been normalized to WT-CFTR in DMSO (ctrl) at the 0-h (0′) chase for each experiment. Data in *C*, *E*, and *F* have been normalized to WT-CFTR in DMSO (ctrl) at the 2-h (120′) chase for each experiment. No samples at the 0-h chase contained N2a, and this time point has not been normalized. *One data point/condition was removed from lanes where the signal/noise ratio was less than 1.5× background. N1a, protease-resistant NBD1-specific fragment; N2a, protease-resistant NBD2-specific fragment; T1d-f, protease-resistant TMD1-specific fragments; T2c, protease-resistant TMD2-specific fragment. T1, T2, and N2 fragments represent domain assembly, which is a late-folding stage of CFTR.

Quantification of experiments to assess total CFTR, percentage of Golgi-modified form (G), and CFTR domain-specific fragments as shown in Fig. 3 are demonstrated in Fig. 4.

In conclusion, R751L-CFTR was biochemically indistinguishable from WT-CFTR in relation to folding, domain assembly, transport out of the ER to the Golgi complex and cell surface, and responses to VX-809 and VX-770.

## DISCUSSION

In this study, we have provided a description of the disease phenotype associated with R751L. We have combined functional assessment of R751L-CFTR activity with molecular assays to provide the first detailed characterization of this variant. We have shown that the patients harboring the R751L variant have mild disease clinically and that R751L-CFTR is biochemically indistinguishable from WT-CFTR.

### R751L-CFTR Is Associated with a Mild Clinical Phenotype

All three individuals in this study had a mild clinical phenotype, as reflected by lung function, nutritional status, and evidence of pancreatic exocrine sufficiency. This is in contrast to the severe phenotype of pulmonary dysfunction and pancreatic insufficiency classically associated with F508del (resulting in defective protein folding) and G551D (resulting in defective channel gating) when arising in conjunction with another severe disease-causing mutation (2). Notably, *patient 1* (F508del/R751L) had an increase in respiratory symptoms compared with *patients 2* and *3* (G551D/R751L) and isolated *B. cepacia* and *P. aeruginosa*. These organisms are typically associated with poorer respiratory outcomes in CF but did not impact upon *patient 1*’s lung function (2). This described clinical phenotype is comparable with that seen with other mutations associated with pancreatic sufficiency and residual CFTR function, including the class IV R117H (p.Arg117His) and class V 3849 + 10kbC->T mutants (23). Notably, all three individuals had elevated sweat chloride levels, and this has been discussed in detail below.

### CFTR-Mediated Chloride Secretion Was Reduced in F508del/R751L HBEs

Chloride transport was reduced in F508del/R751L compared with non-CF HBEs, but to a lesser degree than in F508del/F508del HBEs. Importantly, this differed to our *I_M_* assessment of R751L-CFTR-expressing oocytes and biochemical evaluation in HEK-293 cells. It is possible that a “normal” CFTR current arising from R751L in conjunction with the presence of dysfunctional F508del could explain the reduction in CFTR function seen in the F508del/R751L HBEs relative to non-CF HBEs.

Short-circuit current response to CFTR_inh_-172 in F508del/R751L HBEs was 15% greater than the forskolin-induced *I_sc_* response. CFTR_inh_-172 is a potent CFTR inhibitor, and this finding suggests that there was some constitutive CFTR activity in these cultures. Alternatively, CFTR_inh_-172 may inhibit chloride secretion by a CFTR-independent mechanism, including inhibition of alternative chloride channels, which requires further investigation (24).

Samples from one patient source are not sufficient to draw firm conclusions; however, there are no detailed published clinical or in vitro data on the R751L-CFTR mutation preceding our study. Our work was performed in accordance with existing ethical approval and available culture material sampled at clinical bronchoscopy. In the absence of available cultures from *patients 2* and *3*, we chose to compare *I_sc_* responses of F508del/R751L HBEs with non-CF and F508del/F508del HBEs. Notably, the observed inter-donor variability of *I_sc_* responses in non-CF HBEs paralleled findings in other studies and provided a useful comparator group (25).

### Molecular and Functional Properties of R751L-CFTR Are Similar to WT-CFTR

In contrast to our work in HBEs but in keeping with the clinical phenotype, our functional and molecular characterization of R751L-CFTR demonstrated that *1*) the functional properties of R751L-CFTR were similar to WT-CFTR, *2*) CFTR transport to the cell surface and CFTR folding were not compromised by R751L, and *3*) the biochemical responses of R751L-CFTR to both CFTR modulators VX-770 and VX-809 were indistinguishable from those seen with WT-CFTR.

The total CFTR conductance of a cell is a product of channel number, open probability, and single-channel conductance (26); our *I_M_* measurements found that this parameter was not different between R751L and WT-CFTR. Because our biochemical data demonstrated similar cell surface expression of WT and R751L-CFTR, this suggests that channel number was not affected by the mutation. The response to the CFTR potentiator genistein was also not different between WT and R571L, suggesting that channel gating might not be defective. However, response to a potentiator alone cannot tell whether the absolute open probability is fundamentally different. Specific investigation of any impact of R751L-CFTR on open probability and/or channel conductance would require single-channel recordings using the patch clamp technique, which was not possible in the context of this work and is a limitation of this study (27). However, R751L-CFTR may not affect single-channel conductance due to its specific location in the R domain, which is not within the anion permeation pathway of CFTR in its open state (28).

### Comparison of R751L-CFTR with Other Mutations in the R Domain

We hypothesized that the specific location of R751L and its proximity to key phosphorylation sites, such as S753, could influence CFTR channel function, including channel gating and open probability. Assessing forskolin concentration responses in *Xenopus* oocytes could have further elucidated this; however, recognized transient and variable forskolin responses in oocytes would limit the utility of this experimental approach and ability to draw firm conclusions. Responses to the CFTR potentiator genistein in our experimental work did not differ between R751L-CFTR and WT-CFTR expressing oocytes, which supports the possibility that R751L-CFTR may not affect channel gating. We do, however, acknowledge that genistein application in this context is unlikely to have revealed differences in phosphorylation. Furthermore, although PKA sites are highly conserved, the R domain is considered the least conserved region in CFTR (8). Previous work has shown that complete removal of the R domain has a minimal effect on CFTR gating, and removal of R domain residues from 708–759 (Δ708–759-CFTR) generated similar transepithelial chloride currents to WT-CFTR (29, 30). The Δ708–759-CFTR variant would have incorporated deletion of the consensus phosphorylation site S753, suggesting that not all phosphoserines are required for CFTR channel activity.

Although not abundant, missense mutations within the R domain have varying implications. One study found a clear separation between mutations within the highly conserved NH_2_-terminal region of the R domain that affected CFTR processing to the cell membrane with those in proximity to consensus phosphorylation sites (such as R751L) within the COOH terminus that did not affect CFTR maturation but had varying effects on channel function, including reduced (e.g., R792G) or no effect on chloride channel activity (e.g., R766M) (31). Other neighboring mutations can be associated with varying degrees of pancreatic sufficiency. These include L619S, which affects chloride channel function, and the D614G and I618T variants that show partial channel function due to altered rates of opening. Although first ascribed to arise in proximity to the NH_2_ terminus of the R domain, more recent cryo-EM modeling has shown that these mutations are localized in NBD1 (32, 33). Another interesting variant is T2562G, which affects CFTR function and stability through its effects on the Thr^854^ residue (again in proximity to R751L). Although not CF causing, T2562G is associated with CFTR-related disorders (34); however, recent CFTR modeling has shown that it is located in TMD2 (32).

There is little evidence to suggest that missense mutations within the R domain significantly affect CFTR function, as supported by our findings with R751L. Although R751 is the site of two other variants (R751C and R751P), and despite their association with mild respiratory disease, detailed information regarding these variants is still lacking.

### Interpretation of Elevated Sweat Chloride Values Associated with R751L-CFTR

All three study individuals had abnormally elevated sweat chloride values (>60 mmol/L). Notably, the mean sweat chloride in our patients (96 mmol/L) was greater than values seen in two previous studies of pancreatic sufficient patients (73 and 85 mmol/L) and more comparable with pancreatic insufficient patients (102 mmol/L in both) and our study F508del/F508del individuals (104 mmol ±6) (23, 35). CFTR genotype is strongly correlated with abnormal sweat chloride levels despite its weaker correlation with clinical outcome (36, 37). The presence of F508del or G551D as the second allele in our individuals with R751L could have influenced elevated sweat chloride and contributed to reduced Cl^−^ transport in F508del/R751L HBEs.

Alternative mechanisms for elevated sweat chloride in the study individuals must also be considered, including the possibility of reduced ENaC function. In the healthy sweat duct, the combined actions of ENaC and CFTR result in net sodium chloride absorption (26). A reduction in ENaC-mediated sodium absorption would result in reduced chloride absorption and an increase in sweat chloride. The F508del/R751L HBEs in our work did indeed show a smaller *I_sc_* response to amiloride, suggesting reduced ENaC function, but this finding does require further investigation. Importantly, ENaC mutations have been identified in people with one or no *CFTR* mutations, and heterozygosity of CFTR and ENaC mutations have been associated with dysfunctional ion transport and diffuse bronchiectasis (38, 39). Type 1 systemic pseudohypoaldesteronism (PHA) is also caused by ENaC mutations and is associated with elevated sweat chloride and lower respiratory tract infections (40–42). However, this diagnosis is unlikely in our study individuals due to the absence of other symptoms typically associated with type 1 PHA, including salt-wasting crises characterized by dehydration and failure to thrive early in life (40, 43). Other external factors, including the presence of alternative chloride channels, may contribute to elevated sweat chloride (5). Notably, this latter notion could also be supported by the relatively larger CFTR_inh_-172-sensitive *I_sc_* response in F508del/R751L HBEs compared with the forskolin-stimulated *I_sc_*.

### Concluding Remarks

This report of R751L-CFTR combines clinical phenotype with associated functional and biological implications. It will help inform approaches toward patient follow-up, surveillance, and counseling for parents with newly diagnosed infants with R751L-CFTR. Information regarding the mild associated phenotype will prevent undue anxiety in a situation where the outlook was previously uncertain. Furthermore, it will build upon existing knowledge of mutations within this specific region of the R domain. There is a significant unmet need to characterize rare mutations in the present evolving era of CFTR modulator development to importantly guide their appropriate application.

Our findings suggest that R751L-CFTR is associated with similar functional properties to wild-type CFTR. Alternative factors may be responsible for the clinical features in our study patients, specifically elevated sweat chloride and the suggestion of reduced ENaC function, which may not be adequately explained by dysfunctional CFTR alone. Our work has importantly raised the possibility that alternative molecular defects involving ENaC or alternative chloride channels may be implicated, and further studies are required to elucidate this further.

## DATA AVAILABILITY

Data created in this research are openly available at the Newcastle University Data Repository (https://ncl.ac.uk).

## GRANTS

This work was supported by a Wellcome Trust Clinical Research Training Fellowship [203520/Z/16/Z] to I.J.H; a Medical Research Council Clinician Scientist Fellowship [MR/M008797/1] to M.B; CF Trust Strategic Research Center grants [SRC003] and [SRC013] to M.A.G., C.W., and M.B.; the Netherlands Organization for Health Research and Development [ZonMw TOP 40-00812-98-14103], Dutch Research Council [LIFT 731.017.404], and CFF [BRAAKM14XX0] for the P.vdS.-I.B. Laboratory. The research was supported by the National Institute for Health Research (NIHR) Newcastle Biomedical Research Center based at Newcastle Hospitals NHS Foundation Trust and Newcastle University.

## DISCLAIMERS

The views expressed are those of the authors and not necessarily those of the NHS, the NIHR, or the Department of Health.

## DISCLOSURES

M.B. has received investigator-led research grants from Pfizer and Roche Diagnostics; speaker fees were paid to Newcastle University from Novartis, Roche Diagnostics, and TEVA and travel expenses from Boehringer Ingelheim and Vertex Pharmaceuticals. C.O. has received expenses from Fisher and Paykel and speaker fees from Abbvie. None of the other authors has any conflicts of interest, financial or otherwise, to disclose.

## AUTHOR CONTRIBUTIONS

I.J.H., I.B., C.W., M.A.G., M.B. and M.A. conceived and designed research; I.J.H., M.A., H.Y.Y. and U.J. performed experiments; I.J.H., M.A., H.Y.Y. and U.J. analyzed data; I.J.H., P.v.d.S., I.B., C.W., M.A.G., M.B., M.A., A.I.G., H.Y.Y., U.J., V.S. and B.V. interpreted results of experiments; I.J.H., M.A., H.Y.Y. and U.J. prepared figures; I.J.H. and M.A. drafted manuscript; I.J.H., M.B., M.T., S.B., P.v.d.S., I.B., C.W., M.A.G., M.B., M.A., A.I.G., H.Y.Y., U.J., V.S., B.V., J.T. and C.O. edited and revised manuscript; I.J.H., I.B., C.W., M.A.G., M.B. and M.A. approved final version of manuscript.
